# CDP-choline accumulation in breast and colorectal cancer cells treated with a GSK-3-targeting inhibitor

**DOI:** 10.1007/s10334-018-0719-3

**Published:** 2018-11-16

**Authors:** Su Myat Phyu, Chih-Chung Tseng, Tim Andrew Davies Smith

**Affiliations:** Biomedical Physics Building, School of Medicine, Medical Sciences and Nutrition, University of Aberdeen, Foresterhill, Aberdeen, AB25 2ZD UK

**Keywords:** Glycogen synthase kinase 3, CDP-choline, ^31^P nuclear magnetic resonance spectroscopy, Lipid, Glucose, Choline

## Abstract

**Purpose:**

Glycogen synthase kinase 3 (GSK3) is a key controlling element of many cellular processes including cell-cycle progression and recent studies suggest that GSK3 is a potential anticancer target. Changes in glucose metabolism associated with GSK3 inhibition may impact on lipid synthesis, whilst lipid metabolites can act as molecular response markers.

**Methods:**

Here, SKBr3 breast and HCT8 colorectal cancer cells were treated with the GSK3 inhibitor SB216763, and [^14^C (U)] glucose and [^3^H] choline incorporation into lipids was determined. Cell extracts from treated cells were subject to ^31^P NMR spectroscopy.

**Results:**

SB216763 treatment decreased choline incorporation into lipids and caused an accumulation of CDP-choline which was accompanied by decreased conversion of glucose into lipid components.

**Conclusion:**

SB216763 profoundly inhibits phospholipid synthesis in cancer cells which demonstrate accumulation of CDP-choline detectable by ^31^P NMR spectroscopy. Metabolic changes in lipid metabolism present potential response markers to drugs targeting GSK3.

## Introduction

Glycogen synthase kinase 3 (GSK3) is a family of serine/threonine kinases comprising GSK3α and GSK3β which was originally identified as a key controller of metabolism via its inhibitory phosphorylation of glycogen synthase [1]. More recently, GSK3 has been shown to control a broad range of cellular functions through phosphorylation of many target proteins [2]. These functions include cell-cycle progression, energy homeostasis and inflammation [2]. GSK3 activity is constitutively activated and control of its activity is chiefly through inhibitory phosphorylation of serine-9. One major pathway responsible for this inhibition is the PI3K/PDK1/Akt pathway [3] which is frequently up-regulated in cancer and controls glucose metabolism [4]. Abnormal GSK3 activity is associated with several disease states including diabetes, inflammation and cancer [5]. It has been proposed that GSK3 could be a target for anticancer treatments [6] and studies have shown that inhibition of GSK3 activity is associated with suppressed cancer growth, and in breast and colorectal cancer, it can attenuate resistance to anticancer treatments [7]. Inhibition of GSK3 in glioma has been shown to induce cell death preceded by changes in glucose metabolism including reduced glucose content [8].

Cancer is associated with increased phospholipid metabolism due to high demand for membrane synthesis and intracellular signalling. ^31^P NMR spectroscopy is an analytical technique which facilitates quantitation of phosphorus-containing molecules in cells, tissues and their extracts [9, 10]. The technique is clinically translatable and patient studies of cancer tissue in vivo and subsequent analysis of extracts of the tumour have demonstrated high levels of the phosphatidylcholine metabolite phosphocholine (PCho) [11] which decrease in cancers responding to treatment [12].

As phospholipid synthesis requires intermediates from glucose metabolism, we hypothesised that inhibition of GSK3 may result in changes in phospholipid metabolite content that may be evident in using ^31^P NMR. To investigate this, we have subjected chemical extracts from cancer cells treated with growth inhibitory doses of the GSK3 inhibitor SB216763 to ^31^P NMR spectroscopy.

## Methods

### Materials

Unless otherwise stated, all chemicals were purchased from Sigma-Aldrich (Poole Dorset). Cancer cell lines were purchased from ATCC (LGC Standards, Teddington, UK) and maintained in Dulbecco’s Modified Eagles medium (Gibco, Life Technologies ltd, UK) supplemented with 10% foetal bovine serum and penicillin (100µU/ml)/streptomycin (100 µg/ml) in 75 cm^2^ tissue culture flasks until confluent. MDA-MB-468 breast cancer cells and HCT-8 colorectal cancer cells were obtained from the American Tissue Culture Collection (ATCC) (LGC standards UK).

### Cytotoxicity assay

The sensitivity of cancer cells to the GSK3 inhibitor SB216763 was determined using the MTT [(3-(4,5-Dimethylthiazol-2-yl)-2,5-Diphenyltetrazonium Bromide] assay. Briefly, cells were seeded in 96-well plates by adding 100 µl of cells per well at cell densities of 5 × 10^4^ per ml. The cells were left overnight at 37 °C in a CO_2_ incubator, and then treated with a range of concentrations of SB216763 (1–200 µM) for 72 h. Cells were then quantified by addition of MTT (0.5 mg/ml in medium) and incubation for 2 h followed by removal of medium and addition of DMSO (200 µl). The absorbance was then measured at 570 nm using a scanning multi-well spectrophotometer (Dynatech MR 5000, Dynatech Laboratories, Chantilly,VA, USA) after 10-s agitation and the readings analysed using Ascent software. IC_50_s were calculated using CompuSyn software (ComboSyn, Paramus, NJ, USA).

### [^3^H-methyl]Choline incorporation assay

Cells (10^6^) in 5 ml of medium were seeded in eight 25 cm^2^ tissue culture flasks and left overnight in a CO_2_ incubator at 37 °C. The cells were then treated for 4, 24 or 72 h with SB216763 (40 µM) after which the medium was removed and fresh media (0.5 ml) containing 37KBq/ml of [^3^H-methyl] choline were added and incubated at 37 °C for 15 min. The cells were then washed 4 × by the rapid rinsing of each flask with ice-cold phosphate-buffered saline (5 ml). The cells were then incubated with medium (1 ml) for 1 h at 37 °C after which the medium was retained for measurement of radioactivity and the cells detached and transferred into a microfuge tube. The cells were pelleted by centrifuging at 400*g* for 5 min and the supernatant retained for radioactive counting. The cells were then lysed by addition of 0.375 ml of a 1:2 mixture of chloroform and methanol, and periodic agitation on a vortex mixer for 1 h. Then, 0.125 ml of TRIS: HCl buffer (1 mM pH 7.4) and 125 ml of chloroform were added. After shaking the mixture was centrifuged at 10,000*g* for 10 min and the upper (aqueous phase) and lower (lipid phase) were collected. The cell residue at the interface was dissolved in 0.5 ml of NaOH (1 M) and after neutralising with HCl (1 M) the protein content in the cell residue determined. The lipid phase was added to scintillation fluid (5 ml). The volume of the aqueous phase was determined and 100 µl was added to a scintillation vial containing 5 ml of scintillation fluid. A further 100 µl was subject to phosphate precipitation [13] by addition of 100 µl each of Ba(OH)_2_ (0.3 M) and ZnSO_4_ (5%) followed by vigorous shaking for 5 min. The precipitate was pelleted and 150 µl was added to a scintillation vial with 5 ml of scintillation fluid.

### [U-^14^C]Glucose incorporation into lipids and glycerol assay

As described previously [14], cells were prepared as for ‘[^3^H] choline incorporation assay’ and cells were incubated with [^14^C-U] glucose in medium (0.5 ml) (3.7 KBq/ml) for 2 h. The cells were then washed 4 × with ice-cold PBS, detached with trypsin and transferred to a 1-ml microfuge tube and pelleted by centrifugation at 400*g* for 5 min. The supernatant was removed and radioactivity was determined by adding to a scintillation vial containing 5 ml of scintillation fluid and measuring in a scintillation counter. The pelleted cells were suspended in PBS (1 ml), pelleted and radioactivity determined in the wash. The lipid and aqueous components were then fractionated as described for ‘[^3^H-methyl] choline incorporation assay’. The lipid phase volume was measured and radioactivity determined in a 50-µl sample. The remainder was dried at 37 °C and the complex glycerides were subject to saponification by dissolving in a 1:10 water: ethanol solution of KOH (10 M) and heating to 70 °C for 20 min [15]. The glycerol and fatty acids were separated by fractionation into aqueous and lipid phases as described above [16] and the radioactivity was determined in the two phases.

### NMR sample preparation and ^31^P NMR spectroscopy

Cells (2 × 10^6^) were seeded in each of four 75 cm^2^ tissue culture flasks and allowed to achieve 80% confluence in a CO_2_ incubator at 37 °C. Media were then removed and 10 ml of medium containing SB216763 (40 µM) was added to 2 flasks and 10 ml of medium containing the same volume of DMSO (vehicle—typically 10 µl) was added to the two control flasks. After the respective treatment period (24 or 72 h), the cells were detached with trypsin (5 ml) and the trypsin neutralised with ice-cold medium decanted into 15 ml tubes and placed on ice for 10 min. The cells were then centrifuged at 400*g* for 5 min and after removal of the medium, the cells were suspended in ice-cold saline (0.9%) (1 ml) transferred to a microfuge tube, centrifuged at 400*g* for 5 min. This step was repeated twice to remove extracellular phosphate. The pellet was suspended in 0.375 ml of chloroform: methanol (1:2) and 10 µl EDTA (10 mM) and 20 μl of 1-aminopropyl phosphoric acid (0.3 µmol) as an internal standard and left on ice for 1 h with occasional mixing. The aqueous and lipid phases were then separated by addition of 125 ml each of chloroform and TRIS buffer (10 mM pH7.4) followed by centrifugation at 12,000*g* for 10 min. The aqueous phase was then made up to 0.6 ml by addition of 60 µl of D_2_O and water. The NMR measurement was carried out on a Bruker ADVANCE III 400 NMR spectrometer (400 MHz) operating at 161.98 MHz for ^31^P with continuous broadband ^1^H decoupling (WALTZ16) and at least 20,000 acquisitions. The parameters for each acquisition were: acquisition time 1.258 s; relaxation delay 2 s (inter-pulse time = 3.258 s) (metabolite concentrations were the same when spectra were obtained with a 1-s relaxation delay, indicating that metabolites were fully relaxed in this solvent system after a 1-s delay [17]); pulse angle 45°. All acquisitions were made at 25 °C. Metabolite concentrations were determined by comparison with the internal standard (0.3 µmol) signal at 11.8 ppm. The reference for 0 ppm is 85% H_3_PO_4_.

### Glycogen synthesis

Cells were seeded (10^6^ per flask) in 25 cm^2^ flasks, then treated in triplicate when 70% confluent with SB216763. Glycogen synthesis was assessed by measuring conversion of [^14^C-(U)]-glucose into [^14^C-(U)]-glycogen in the cells treated with SB216763 for 4 h. The cells were incubated with 0.25 ml of [^14^C]-glucose (55 KBq/ml) for 90 min, washed rapidly with ice-cold PBS, then dissolved in 150 µl of KOH 20% (w/v) for 1 h at 70 °C. Glycogen was precipitated by addition of 50 µl of glycogen (2 mg/ml) as carrier followed by 0.5 ml of ice-cold ethanol and leaving for 2 h at − 20 °C. After centrifugation at 10,000*g* for 10 min, the supernatant was removed, and the precipitated glycogen dissolved in 150 µl of hot water, and transferred to scintillation vials containing 5 ml of scintillation fluid. The radioactive glycogen value was normalised to protein content carried out on 50 µl of the supernatant after neutralising the KOH with HCl (1 M).

### Statistics

Data are expressed as mean ± SEM and statistical differences determined by the student *t* test and values expressed as (*t* = *x*, *p* < *y*), where *t* values and probability are unitless values. All experiments were carried out 2 or more times.

## Results

### Cancer cell sensitivity to SB216763

**Fig. 1:**
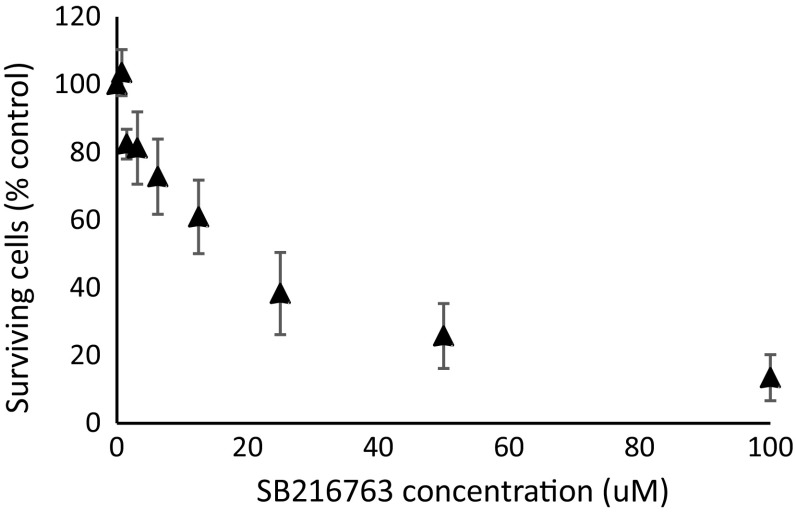
Relative growth of SKBr3 cells treated with doses of 0–100 µM SB216763 for 3 days

The effect of 3-day treatment with different concentrations of SB216763 (0–100 µM) on the growth of SKBr3 cells is shown in Fig. 1. Subsequent experiments were carried out using 40 µM concentrations of SB213673.

### Changes in ^31^P NMR detectable metabolites by SB216763 treatment

**Fig. 2:**
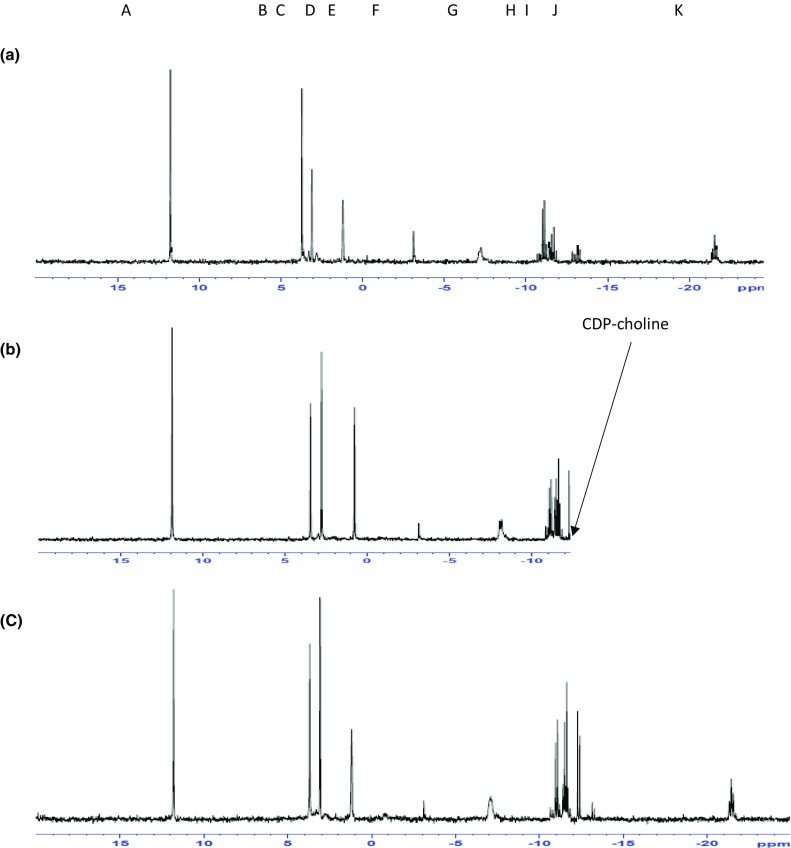

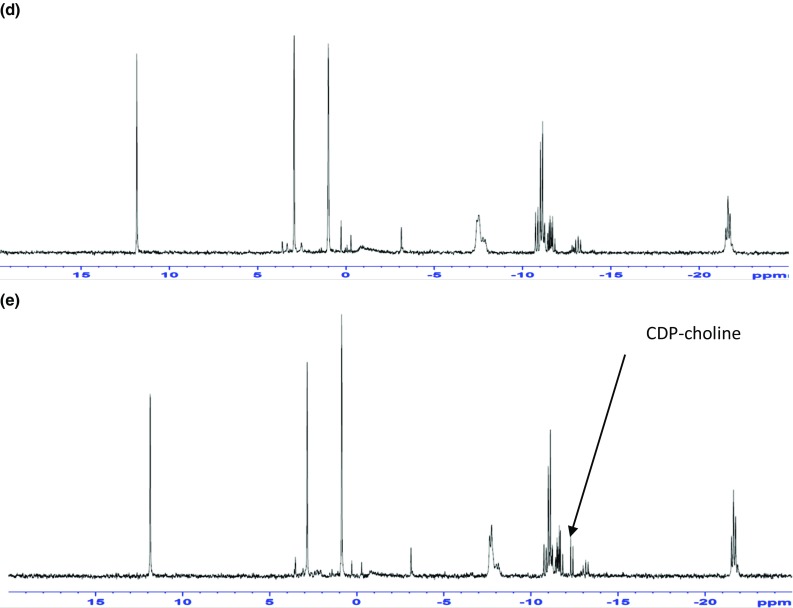
^31^P NMR spectra from extracts of untreated SKBr_3_ cells (**a**) or treated with SB216763 24 h (**b**) or 72 h (**c**) and untreated HCT8 cells (**d**) or treated with SB216763 for 24 h (**e**). Identities of metabolites are shown in Spectrum b (A: AMPA (standard), B: PCho, C: inorganic phosphate D: GPE and GPC (two small peaks) E: Phosphocreatine F: γATP G: αATP H: nicotinamide adenine dinucleotide (NAD species) I: CDP-choline J: NAD species K: βATP

Control cells and cells treated with SB216763 were subject to ^31^P NMR spectroscopy. Figure 2a–c shows an example of ^31^P NMR spectra from a chemical extract of control and SB216763-treated SKBr3 cells. A peak corresponding with CDP-choline which was at very low levels or undetectable levels in the controls was present at significantly higher levels (*p* < 0.05) in the cells treated with SB216763 for 24 h [control 0.002 µmol/mg protein (± 0.001), SB-treated 0.012 µmol/mg protein (± 0.003)] and was also present in cells treated for 72 h (Fig. 2c). The CDP-choline peak was also present in HCT8 colorectal cancer cells treated with SB216763 for 24 h (Fig. 2e) but not controls (Fig. 2d). CDP-choline is formed by reaction of phosphocholine (PCho) with cytidine triphosphate (CTP). The content of PCho was similar in control [0.1 (± 0.012) µmol/mg protein] and SB-treated [0.085 (± 0.03) µmole/mg protein] SKBr3 cells. The next step is the reaction of CDP-choline with diacylglycerol (DAG). As DAG can be formed, de novo, from the glycolysis intermediate glycerol 3 phosphate, the effect of SB216763 on carbon flux from glucose was examined.

### Flux of [^14^C] from glucose to lipids

**Fig. 3:**
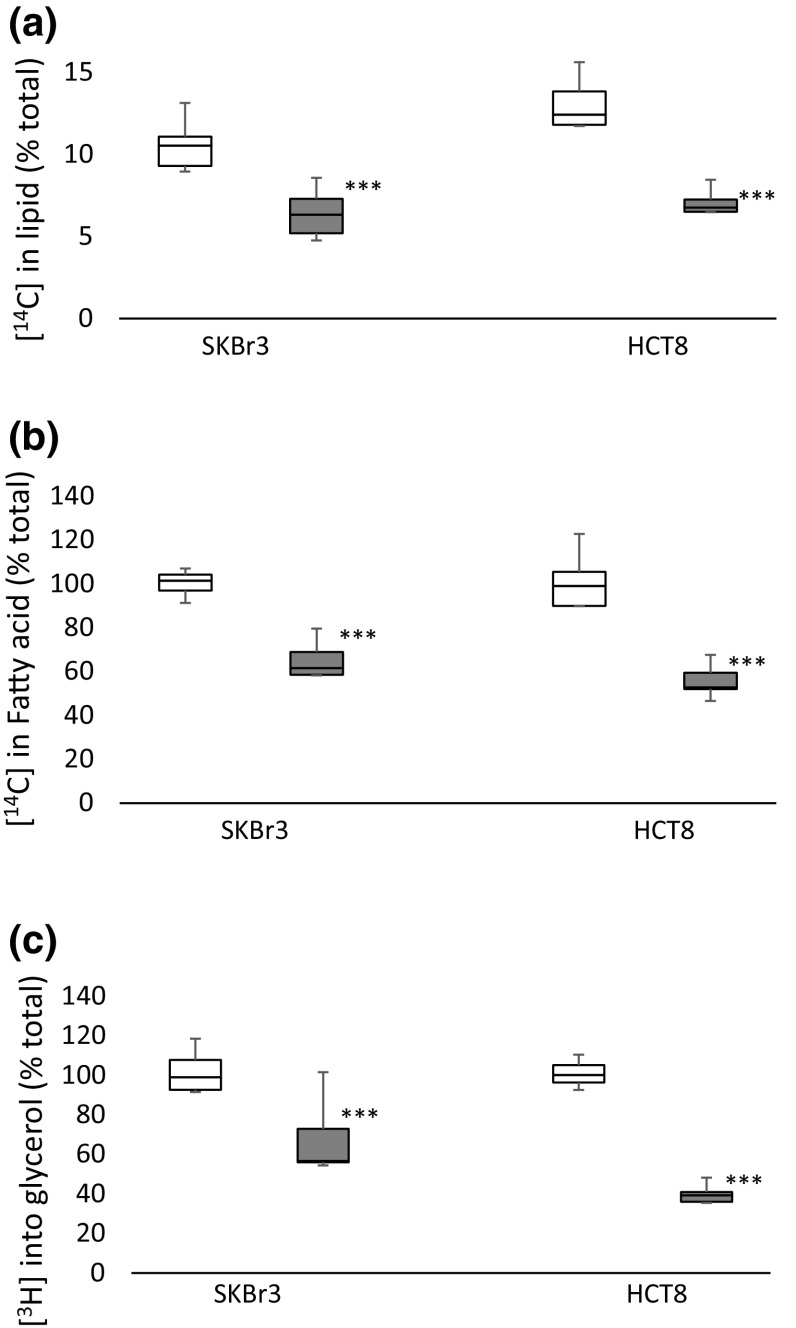
Incorporation of ^14^C into lipid-soluble metabolites (fraction of total [^14^C] glucose uptake) (**a**) and fraction of lipid-soluble ^14^C incorporated into Fatty acids (**b**) and glycerol (**c**) untreated and SB216763-treated SKBr3 and HCT8 cells. Cells were incubated with [^14^C (U)] glucose for 2 h then washed rapidly with ice-cold PBS. Cells were fractionated into aqueous and lipid soluble components and ^14^C-associated with the lipid fraction determined (**a**). The lipid fraction was subject to saponification to release the fatty acids from glycerol and then fractionated into lipid (fatty acids) (control (grey) SB216763-treated (grey)) and aqueous (glycerol) (control (white) SB216763-treated (grey)) and ^14^C-associated with the two fractions determined

Glucose incorporation into lipids was determined in SKBr3 and HCT8 cells. Figure 3 shows the incorporation of glucose into lipid (Fig. 3a) and fatty acids (Fig. 3b) and glycerol (Fig. 3c) in cells treated with SB216763 relative to untreated cells. In both cell lines, the incorporation of ^14^C- from glucose into lipid was decreased (SKBr3: *t* = 11.7, *p* < 0.001; HCT8 *t* = 8.2, *p* < 0.001) and this decrease was found to be in both the glycerol (derived from dihydroxyacetone phosphate during glycolysis) (SKBr3: *t* = 3.1, *p* < 0.01; HCT8: 7.3, *p* < 0.001) and fatty acid (derived from intermediates of the Krebs cycle) (SKBr3: *t* = 11, *p* < 0.001; HCT8: *t* = 9.3, *p* < 0.001) fractions.

### Effect of SB216763 treatment on choline uptake and incorporation into lipids

**Fig. 4:**
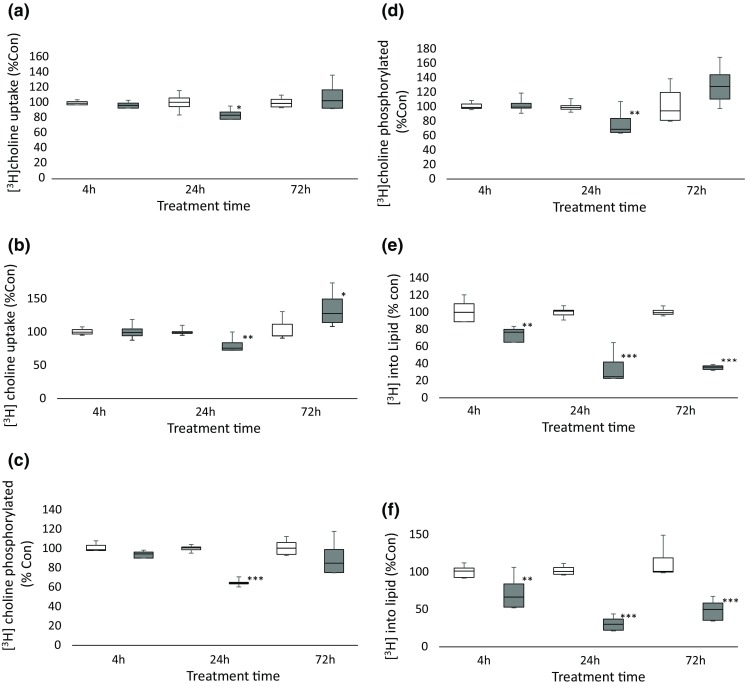
[^3^H-methyl] choline incorporation by untreated (white) and SB2162763-treated (grey) SKBr3 and HCT8 cells. Total uptake (**a**, **b**), phosphorylated fraction (**c**, **d**) and lipid fraction (**e**, **f**). Units: Radioactive counts normalised to mg protein and expressed as a percentage of untreated values (% control). Statistically significant differences indicated by asterisk (*p < 0.05; **p < 0.01; ***p < 0.001)

Figure 4 shows the total uptake (a and b) and proportion of phosphorylated [^3^H-methyl] choline (c and d) and the incorporation into lipids (e and f) by SKBr3 and HCT8 cells treated with SB216763 for 4, 24 and 72 h compared with untreated cells. In each cell line, [^3^H-methyl] choline incorporation into lipids was decreased after 4 h (HCT8: *t* = 5.6, *p* < 0.001; SKBr3: *t* = 16, *p* < 0.001), 24 h (HCT8: *t* = 5.8, *p* < 0.001; *t* = 3.5, *p* < 0.01) and 72 h (HCT8: *t* = 6.6, *p* < 0.001; SKBr3: *t* = 28, *p* < 0.001) treatment with SB216763. The uptake of [^3^H-methyl] choline was unaffected by treatment of cells with SB216763 by each cell line at each time point except HCT8 cells at 72 h which showed a significant (*t* = 3.3 *p* < 0.05) increase in incorporation. The proportion of [^3^H-methyl] choline that was phosphorylated was unchanged in cells treated for 4 h, decreased in HCT8 (*t* = 4.1, *p* < 0.01) and SKBr3 (*t* = 18 *p* < 0.001) cells treated for 24 h and unchanged in cells treated for 72 h with SB216763.

**Fig. 5:**
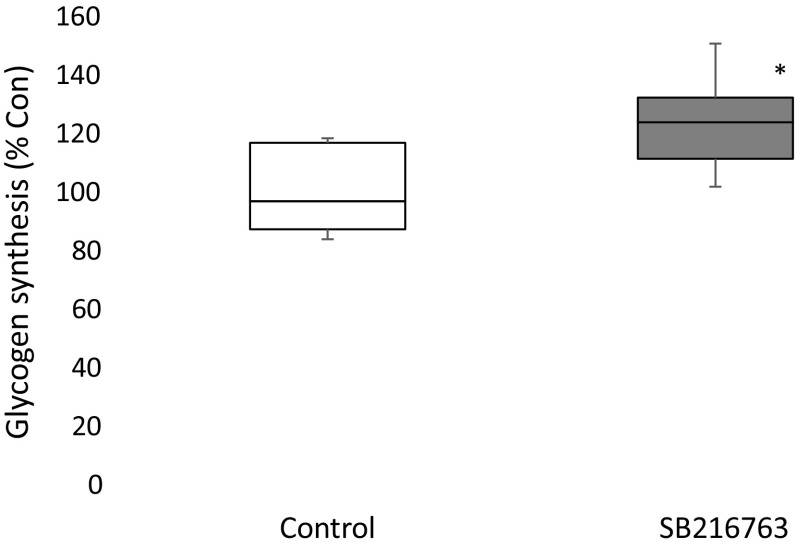
Incorporation of [^14^C] into glycogen by control and SB216763-treated HCT8 cells incubated with [^14^C] glucose

The effect of treatment with SB216763 on glycogen synthesis cells is shown in Fig. 5. Conversion of [^14^C] glucose into [^14^C] glycogen was found to be increased by HCT8 cells treated with SB216763.

## Discussion

De novo synthesis from glucose is the major route for the formation of diacylglycerol (DAG) for metabolic purposes [18]. The flux of carbon from glucose into anabolic pathways responsible for the synthesis of phospholipids is facilitated in cancer cells by decreased oxidative phosphorylation and increased glycolysis resulting in an increased supply of acetyl CoA for glycerol and fatty acid synthesis [19].

Phospholipid metabolism is crucial for proliferation as it provides cell membrane and intracellular signalling components [20]. Phosphatidylcholine (PtdCho) is an abundant phospholipid in mammalian cell membranes [21] and is an important source of intracellular signalling pathway intermediates [22]. Inhibition of PtdCho synthesis in mammalian cells including cancer has been shown to induce cell death [23]. The de novo synthesis of PtdCho occurs by the CDP-choline pathway. Choline, taken into cells by the choline transporter (ChT), is phosphorylated to phosphocholine (PCho) by choline kinase. The enzyme CTP phosphocholine cytidylyltransferase (CT) converts Cytidine triphosphate (CTP) and PCho into CDP-choline. The enzyme CDP-choline:1,2-diacylglycerolcholinephosphotransferase (CPT) then catalyses the conversion of CDP-choline and diacylglycerol into PtdCho.

Breast cancer cells were found to be sensitive to the growth inhibitory effect of the GSK3 inhibitor SB216763 in agreement with others [24]. Zeng et al. [25] have shown that overexpression of GSK3β in non-small-cell lung cancer is associated with poor prognosis and its inhibition decreased cell proliferation by causing cell-cycle arrest in G1/S. They suggested that GSK3β acts as a tumour promoter and a potential therapeutic target. Evidence from work on MCF7 breast cancer cells suggests that the growth inhibitory effect of GSK3β inhibition results subsequent decrease in cyclin D1 expression [26].

Treatment of SKBr3 and HCT8 cells with S216763 decreased the flux of carbon from glucose to lipids and the incorporation of choline into lipids which could be attributed to increased formation of glycogen from glucose associated with GSK3β inhibition [27]. In agreement with these findings Park et al. [28] has recently shown that inhibition of GSK3β in colorectal cancer cells resulted in decreased fatty acid synthesis.

^31^P NMR studies were carried out on cells treated for up to 72 h with SB216763 in common with previous work [29]. Interestingly, CDP-choline formation is considered as the rate-limiting step for PtdCho formation; so, its build up is unusual further suggesting that the decrease in PtdCho in cells treated with SB216763 formation is a consequence of inadequate substrate (DAG) for the final CTP-catalysed reaction of CDP-choline and DAG. Anthony et al. [30] has demonstrated that several drugs induce the cellular build-up of CDP-choline. These drugs included farnesol and chelerythrine which directly inhibit CDP-choline:1,2-diacylglycerol cholinephosphotransferase. They also found that camptothecin and etoposide, which are inhibitors of phosphokinase C and induce apoptosis, also increase CDP-choline accumulation which they attributed to indirect inhibition of CDP-choline:1,2-diacylglycerol cholinephosphotransferase by pH shifts during the apoptotic process. The mechanism for the appearance of CDP-choline in SB216763-treated cells has yet to be fully elucidated but may be a result of  less glucose being utilised in the formation of phospholipid intermediates via glycolysis.

Previous studies [12, 31] have shown that the content of PMEs decrease in tumours responding to treatment and that this may be a useful early response indicator. Although treatment of cancer cells with SB216763 was not accompanied by changes in the concentration of PCho, the appearance of the CDP-choline peak in the ^31^P NMR spectrum suggests that this might be a useful early indicator of response to drugs targeting GSKβ. However, early expectations that in vivo ^31^P NMR spectroscopy could be used in the clinic have not yet materialised, mainly due to the relative insensitivity of the technique compared with, for example [^18^F]-FDG-PET. A further problem with the quantification of the CDP-choline resonance in in vivo spectra is its chemical shift, which is close to that of nucleotide adenine.dinucleotide (NAD(H)) resonance with which it most likely forms a composite peak. On a more positive note, instruments with higher magnetic field strengths and proton decoupling are improving peak resolution [32].

In conclusion, treatment of cancer cells with the GSK3 inhibitor SB216763 results in decreased transfer of carbon from glucose to lipids, cellular accumulation of CDP-choline and decreased incorporation of choline into PtdCho.

## References

[1] Embi N, Rylatt DB, Cohen P (1980). Glycogen synthase kinase-3 from rabbit skeletal muscle. Separation from cyclic-AMP-dependent protein kinase and phosphorylase kinase. Eur J Biochem.

[2] Liu X, Yao Z (2016). Chronic over-nutrition and dysregulation of GSK3 in diseases. Nutr Metab.

[3] Majewski N, Nogueira V, Robet RB (2004). Akt inhibits apoptosis downstream of BID cleavage via a glucose-dependent mechanism involving mitochondrial hexokinases. Mol Cell Biol.

[4] Fleming IN, Andriu A, Smith TAD (2014). Early changes in [18F] FDG incorporation by breast cancer cells treated with trastuzumab in normoxic conditions: role of the Akt-pathway, glucose transport and HIF-1α. Breast Cancer Res Treat.

[5] Eldar-Finkelman H (2002). Glycogen synthase kinase 3: an emerging therapeutic target. Trends Mol Med.

[6] Mccubrey JA, Steelman LS, Bertrand FE (2014). GSK3 as potential target for therapeutic intervention in cancer. Oncotarget.

[7] Domoto T, Pyko IY, Furuta T (2016). Glycogen synthase kinase-3 is a pivotal mediator of cancer invasion and resistance to therapy. Cancer Sci.

[8] Kotliarova S, Pastorino S, Kovell LC (2008). Fine, Glycogen synthase kinase-3 inhibition induces glioma cell death through c-MYC, nuclear factor-kappa B, and glucose regulation. Cancer Res.

[9] Glunde K, Penet MF, Jiang L (2005). Choline metabolism-based molecular diagnosis of cancer: an update. Expert Rev Mol Diagn.

[10] Podo F, Saradanelli E, Iorio R (2007). Abnormal choline phospholipid metabolism in breast and ovary cancer: molecular bases for noninvasive imaging approaches. Curr Med Imaging Rev.

[11] Smith TAD, Glaholm J, Leach MO (1991). A comparison of in vivo and in vitro 31P NMR spectra from human breast tumours: variations in phosphorus metabolism. Br J Cancer.

[12] Leach MO, Verrill M, Glaholm J (1998). Measurements of Human Breast Cancer using Magnetic Resonance Spectroscopy: a review of clinical measurement and a report of localized ^31^P measurements of response to treatment. NMR Biomed.

[13] Kletzien RF, Perdue JF (1974). Sugar transport in chick embryo fibroblasts. J Biol Chem.

[14] Smith TAD, Phyu SM (2016). Metformin decouples phospholipid metabolism in breast cancer cells. PLoS One.

[15] Hosotani K, Kitagawa M (2003). Improved simultaneous determination method of b-carotene and retinol with saponification in human serum and rat liver. J Chromatogr B.

[16] Tan HM, Aziz ARA, Aroua MK (2013). Glycerol production and its applications as a raw material: a review. Renew Sustain Energy Rev.

[17] Phyu SM, Tseng CC, Fleming IN, Smith TAD (2016). Probing the PI3K/Akt/mTor pathway using 31P-NMR spectroscopy: routes to glycogen synthase kinase 3. Sci Rep.

[18] Carrasco S, Merida I (2007). Diacylglycerol, when simplicity becomes complex. Trends Biochem Sci.

[19] Ridgway ND (2013). The role of phosphatidylcholine and choline metabolites to cell proliferation and survival. Crit Rev Biochem Mol Biol.

[20] Arlauckas SP, Popov AV, Delikatny EJ (2016). Choline kinase alpha-Putting the ChoK-hold on tumor metabolism. Prog Lipid Res.

[21] Ide Y, Waki M, Hayasaka T (2013). Human breast cancer tissues contain abundant phosphatidylcholine(36:1) with high stearoyl-CoA desaturase-1 expression. PLoS One.

[22] Sovadinova I, Babica P, Boeke H (2015). Phosphatidylcholine specific PLC-induced dysregulation of gap junctions, a robust cellular response to environmental toxicants, and prevention by resveratrol in a rat liver cell model. PLoS One.

[23] Cui Z, Houweling M (2002). Phosphatidylcholine and cell death. Biochim Biophys Acta.

[24] Trnski D, Sabol M, Gojevic A (2015). GSK3 beta and Gli3 play a role in activation of Hedgehog-Gli pathway in human colon cancer—targeting GSK3 beta downregulates the signaling pathway and reduces cell proliferation. Biochim Biophys Acta Mol Basis Dis.

[25] Zeng J, Liu D, Qiu ZX (2014). GSK3 beta overexpression indicates poor prognosis and its inhibition reduces cell proliferation and survival of non-small cell lung cancer. PLoS One.

[26] Kim HM, Kim CS, Lee JH (2013). CG0009, a novel glycogen synthase kinase 3 inhibitor, induces cell death through cyclin d1 depletion in breast cancer cells. PLoS One.

[27] Ha DT, Trinh NT, Hien TT (2010). Selected compounds derived from Moutan Cortex stimulated glucose uptake and glycogen synthesis via AMPK activation in human HepG2 cells. J Ethnopharmacol.

[28] Park GB, Chung YH, Gong JH (2016). GSK-3 beta-mediated fatty acid synthesis enhances epithelial to mesenchymal transition of TLR4-activated colorectal cancer cells through regulation of TAp63. Int J Oncol.

[29] Al-saffar NMS, Jackson E, Raynaud FI (2010). The phosphoinositide 3-kinase inhibitor PI-103 downregulates choline kinase a leading to phosphocholine and total choline decrease detected by magnetic resonance spectroscopy. Cancer Res.

[30] Anthony ML, Zhao M, Brindle KM (1999). Inhibition of phosphatidylcholine biosynthesis following induction of apoptosis in HL-60 cells. J Biol Chem.

[31] Al-Saeedi F, Welch AE, Smith TAD (2005). [methyl-3H] choline incorporation into MCF7 tumour cells: correlation with proliferation. Eur J Nucl Med.

[32] Lagemaat MW, Maas MC, Vos EK (2015). ^31^P MR spectroscopic imaging of the human prostate at 7T:T1 relaxation times, nuclear overhauser effect, and spectral characterization. Magn Resonan Med.

